# The Metalloprotease Meprin β Is an Alternative β-Secretase of APP

**DOI:** 10.3389/fnmol.2016.00159

**Published:** 2017-01-05

**Authors:** Christoph Becker-Pauly, Claus U. Pietrzik

**Affiliations:** ^1^Unit for Degradomics of the Protease Web, Institute of Biochemistry, University of KielKiel, Germany; ^2^Institute for Pathobiochemistry, University Medical Center of the Johannes Gutenberg-University MainzMainz, Germany

**Keywords:** meprin β, N-terminal truncated Aβ, APP, shedding, proteolysis

## Abstract

The membrane bound metalloprotease meprin β is important for collagen fibril assembly in connective tissue formation and for the detachment of the intestinal mucus layer for proper barrier function. Recent proteomic studies revealed dozens of putative new substrates of meprin β, including the amyloid precursor protein (APP). It was shown that APP is cleaved by meprin β in distinct ways, either at the β-secretase site resulting in increased levels of Aβ peptides, or at the N-terminus releasing 11 kDa, and 20 kDa peptide fragments. The latter event was discussed to be rather neuroprotective, whereas the ectodomain shedding of APP by meprin β reminiscent to BACE-1 is in line with the amyloid hypothesis of Alzheimer's disease, promoting neurodegeneration. The N-terminal 11 kDa and 20 kDa peptide fragments represent physiological cleavage products, since they are found in human brains under different diseased or non-diseased states, whereas these fragments are completely missing in brains of meprin β knock-out animals. Meprin β is not only a sheddase of adhesion molecules, such as APP, but was additionally demonstrated to cleave within the prodomain of ADAM10. Activated ADAM10, the α-secretase of APP, is then able to shed meprin β from the cell surface thereby abolishing the β-secretase activity. All together meprin β seems to be a novel player in APP processing events, even influencing other enzymes involved in APP cleavage.

## Introduction

To date, more than 35,000 research articles dealing with the amyloid precursor protein (APP) are annotated in Pubmed (13.10.2016) and most of these papers are related to Alzheimer's disease. Nevertheless, APP is still an enigma in terms of its physiological and pathophysiological functions.

APP is a multi-domain glycosylated type 1 transmembrane protein. Earlier studies reported that the ectodomains of APP family proteins have zinc- (Bush et al., 1993) and copper binding-properties (Simons et al., 2002) and that APP is able to reduce bound Cu^2+^ to Cu^+^ (Multhaup et al., 1996). Moreover, APP has been proposed to bind extracellular matrix proteins like heparin and collagen (Small et al., 1994), and to have a receptor-like function (Beher et al., 1996). In this context, it became more and more challenging, whether APP can form cellular cis-dimers (Scheuermann et al., 2001), reminiscent of classical receptor dimerization described for the EGF receptor (Schlessinger, 2002). However, there is accumulating evidence from biochemical and structural data that APP can form homodimers (Scheuermann et al., 2001; Kaden et al., 2009; Isbert et al., 2012) as well as heterodimers with its homologs APLP1 and APLP2 (Soba et al., 2005).

To date, at least three domains have been reported to promote APP dimerization: first the E1 domain containing the N-terminal Growth factor like domain (GFLD) and Copper binding domain (CuBD) (Soba et al., 2005). The second dimerization interface is represented by the E2 domain (amino acids 365–699), the largest subdomain of the APP ectodomain, containing the carbohydrate- and the juxtamembrane region. Crystallographic and X-ray structure modeling revealed that the E2 region can reversibly dimerize in an antiparallel orientation in solution (Wang and Ha, 2004) and it has been reported that binding of extracellular matrix components, such as heparin, to this domain may also regulate dimerization (Gralle et al., 2006). However, in contrast to Wang and colleagues a study by Dulubova and colleagues could not confirm that the E2 domain does dimerize in solution (Dulubova et al., 2004). A third dimerization interface is located at the extracellular juxtamembrane/transmembrane (JM/TM) boundary, where APP contains three consecutive glycine-xxx-glycine (GxxxG) motifs (Munter et al., 2007; Gorman et al., 2008; Kienlen-Campard et al., 2008) one embedded within the Aβ sequence.

Interestingly, detection of APP dimerization *in vivo* showed a possibility that the efficient processing of APP by α- and β-secretases (see below) may depend on its oligomerization state that results in cooperative effects for these allosteric enzymes (Schmidt et al., 2012).

Although the German psychiatrist Alois Alzheimer was the first to demonstrate a relationship between specific cognitive changes, neurological lesions in the human brain, and clinical history (Alzheimer, 1907), much later the amyloid cascade hypothesis attributed these observations to the presence of the cleavage products of APP in the brain (Hardy and Selkoe, 2002). Alzheimer reported the results of an autopsy on a 55-year-old woman named Auguste Deter and noted the presence of two distinct pathological lesions in Deters brain, which now define Alzheimer's disease (AD): first, the neurofibrillary tangles (NFTs), which accumulate intraneuronal (later shown to be composed of paired helical filaments (PHFs) containing the microtubule-associated protein tau; Goedert et al., 1988, 1989); second, extracellular amyloid deposits in the form of diffuse or neuritic senile plaques (Price et al., 1997). Senile plaques accumulate extracellular and were isolated and purified in 1984 by Glenner and Wong, who showed that it was a ~4 kDa peptide (Aβ), primarily 40 or 42 amino acids in length, which they speculated was cleaved from a larger precursor (Glenner and Wong, 1984). Subsequently, it has been demonstrated that this peptide fragment originated from a larger precursor protein, named the amyloid-β precursor protein (AβPP, or APP as used here) and was characterized from the analysis of a full-length cDNA encoding a translational product of 695 residues (Kang et al., 1987).

## Conventional APP processing

**Figure 1:**
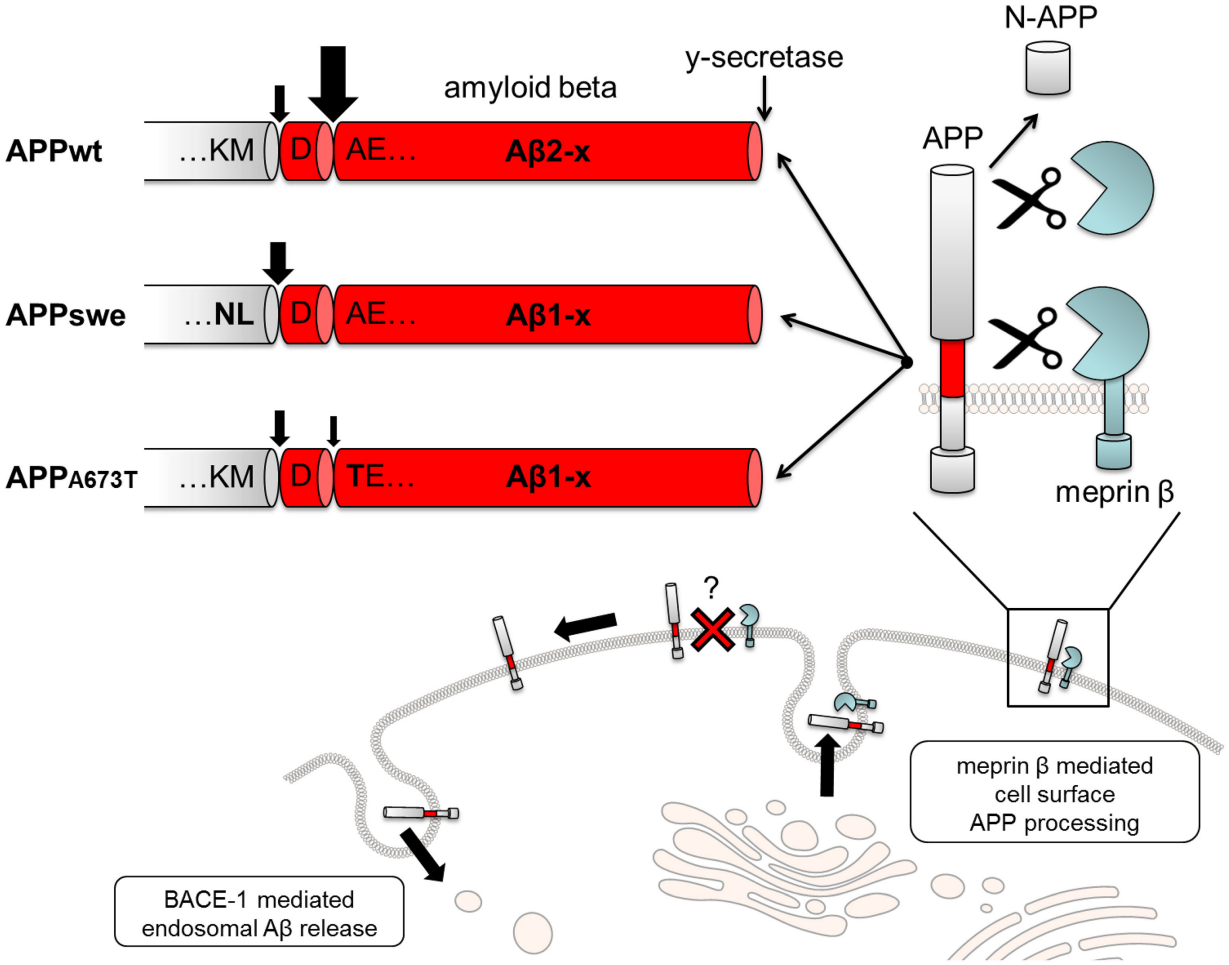
**Proteolytic processing of APP by Meprin β**. APP is cleaved by meprin β in two distinct ways. On the one hand non-amyloidogenic N-APP fragments are produced, and on the other hand, meprin β acts as a β-secretase, inducing Aβ2-x generation. Remarkably, APPswe completely abolishes Aβ2-x release. The AD protective mutant APP_A673T_ is also much less cleaved by meprin β.

Multiple enzymes have been shown to process APP during its lifetime. The non amyloidogenic pathway, in which APP is cleaved within the sequence of the amyloid peptide by a generally named enzyme group called α-*secretase*, precludes the formation of the full-length Aβ which is found in the amyloid core of senile plaques (Zheng and Koo, 2006). One other pathway leads to the production of Aβ peptides from its precursor after the initial cleavage by a generally named enzyme group called β*-secretase* (Hussain et al., 1999; Sinha et al., 1999; Vassar et al., 1999; Yan et al., 1999). The first β–secretase identified was then named β-site APP-cleaving enzyme (BACE-1). BACE-1 is a type I membrane-bound aspartyl protease located in the endosomal/lysosomal compartment (Sinha et al., 1999; Vassar et al., 1999). Cleavage of APP by BACE-1 (Vassar, 2002) occurs between methionine 596 and aspartate 597 of APP695 (Figure 1), producing two fragments, the secreted N-terminal ectodomain sAPPβ and a 10 kDa, 99-amino-acid-long fragment C99, encompassing the Aβ peptide and the remaining C-terminal part. The optimal pH of BACE-1 activity is ~4.5, suggesting that the β-site cleavage of APP occurs preferentially in more acidic compartments, such as in endosomes and lysosomes (Vassar et al., 1999).

After α- or β-cleavage, the carboxyl terminal fragments (CTFs) of APP, known as αCTF (C83) and βCTF (C99), respectively, remain membrane-associated and are further cleaved by the γ-*secretase*-complex (Edbauer et al., 2003). The γ-*secretase* is an aspartyl protease complex (Wolfe et al., 1999), which unlike α- and β-*secretases*, acts within the membrane and cleaves APP at multiple sites (Zhao et al., 2004), releasing either, Aβ and intracellular C-terminal domain fragments (ICDs) or p3 and ICDs (Figure 1). This process is called regulated intramembrane proteolysis (RIP) (Brown et al., 2000). However, while the two predominant forms of Aβ and p3 terminate at valine 637 (Aβ40 and p3/40) and alanine 639 (Aβ42 and p3/42) (Haass et al., 1992a), some isolated ICDs are shorter than expected and begin at sites 9–10 amino acid downstream of those residues (Gu et al., 2001).

BACE-1 is described to be the major Aβ generating β-secretase (Hussain et al., 1999; Sinha et al., 1999; Vassar et al., 1999; Yan et al., 1999; Lin et al., 2000). This was convincingly shown when a genetic knock-out of the protease in mice abolished Aβ generation almost completely (Luo et al., 2001; Roberds et al., 2001; Dominguez et al., 2005). In accordance to that, BACE-1 was found to be upregulated in brains of sporadic AD patients (Fukumoto et al., 2002). However, there is strong evidence that certain amounts of Aβ are generated independently of BACE-1. This was supported, when using potent BACE-1 inhibitors *in vitro* and *in vivo* (Asai et al., 2006; Nishitomi et al., 2006; Hussain et al., 2007; Stanton et al., 2007; Sankaranarayanan et al., 2008). Interestingly, some studies showed that by inhibition of Aβ1-x generating β-secretase activity, alternative N-terminally truncated Aβ peptides increase (Haass et al., 1995; Schrader-Fischer and Paganetti, 1996; Takeda et al., 2004; Schieb et al., 2010; Mattsson et al., 2012). Analysis of Aβ species in BACE-1 knock-out mice likewise revealed that the generation of Aβ1-x peptides was completely abolished while N-terminally truncated Aβ variants could still be generated (Nishitomi et al., 2006). These N-terminally truncated Aβ peptides are also found in the cerebrospinal fluid, brain tissue, and human blood plasma (Wiltfang et al., 2001; Lewczuk et al., 2004; Takeda et al., 2004; Güntert et al., 2006; Lewis et al., 2006; Maler et al., 2007; Murayama et al., 2007). Later it was demonstrated that BACE-1 invariably generates two Aβ variants beginning with the aspartate in p1 or p11, therefore other proteases might account for the production of N-terminally truncated peptides (Citron et al., 1995; Vassar et al., 1999). Indeed, heterogeneity of alternative β-secretase cleavage events has been described (Golde et al., 1992; Haass et al., 1992b; Seubert et al., 1992; Busciglio et al., 1993) leading to alternative Aβ peptides other than Aβ1/11-x (Vigo-Pelfrey et al., 1993; Asami-Odaka et al., 1995; Wang et al., 1996), which could also be found in amyloid plaques *in vivo* (Masters et al., 1985; Güntert et al., 2006). It is not clear whether N-terminally truncated Aβ species are generated via cleavage of APP by yet unknown proteases or via truncation of Aβ1-x after its γ-secretase mediated release, e.g., by aminopeptidase A (Sevalle et al., 2009). In contrast to further subsequent cleavage of already released Aβ peptides, Cathepsin B (Hook et al., 2005, 2014; Kindy et al., 2012), S and L (Schechter and Ziv, 2011) have been discussed to be directly involved in Aβ generation, acting as alternative β-secretases. The enzymatic cleavage events of cathepsins on APP are not fully understood since some groups showed that cathepsins are rather involved in Aβ degradation lowering total Aβ burden (Mueller-Steiner et al., 2006; Letronne et al., 2016).

The amyloid peptides Aβ2-40/42 cannot be assigned to BACE-1 activity and are most likely generated due to an alternative β-secretase cleaving APP between 672Asp/673Ala (Wiltfang et al., 2001; Schieb et al., 2010, 2011). Aβ2-x might act as a precursor and can likewise be processed to Aβ3-x by the alanyl-aminopeptidase activity of aminopeptidase N (Hosoda et al., 1998). This is supposed to occur even under physiological conditions due to activity of cortical aminopeptidase N (Kuda et al., 1997; Wiltfang et al., 2001). It was also discussed that N-terminally truncated Aβ peptides arise when Aβ is degraded by a variety of Aβ degrading enzymes e.g., myelin basic protein, neprilysin, and angiotensin-converting enzyme (Saido and Leissring, 2012). But until recently no proof about the exact mechanisms leading to N-terminally truncated Aβ variants could be given, which changed by the identification of the metalloprotease meprin β as an alternative β-secretase described below.

## Alternative APP processing

**Figure 2:**
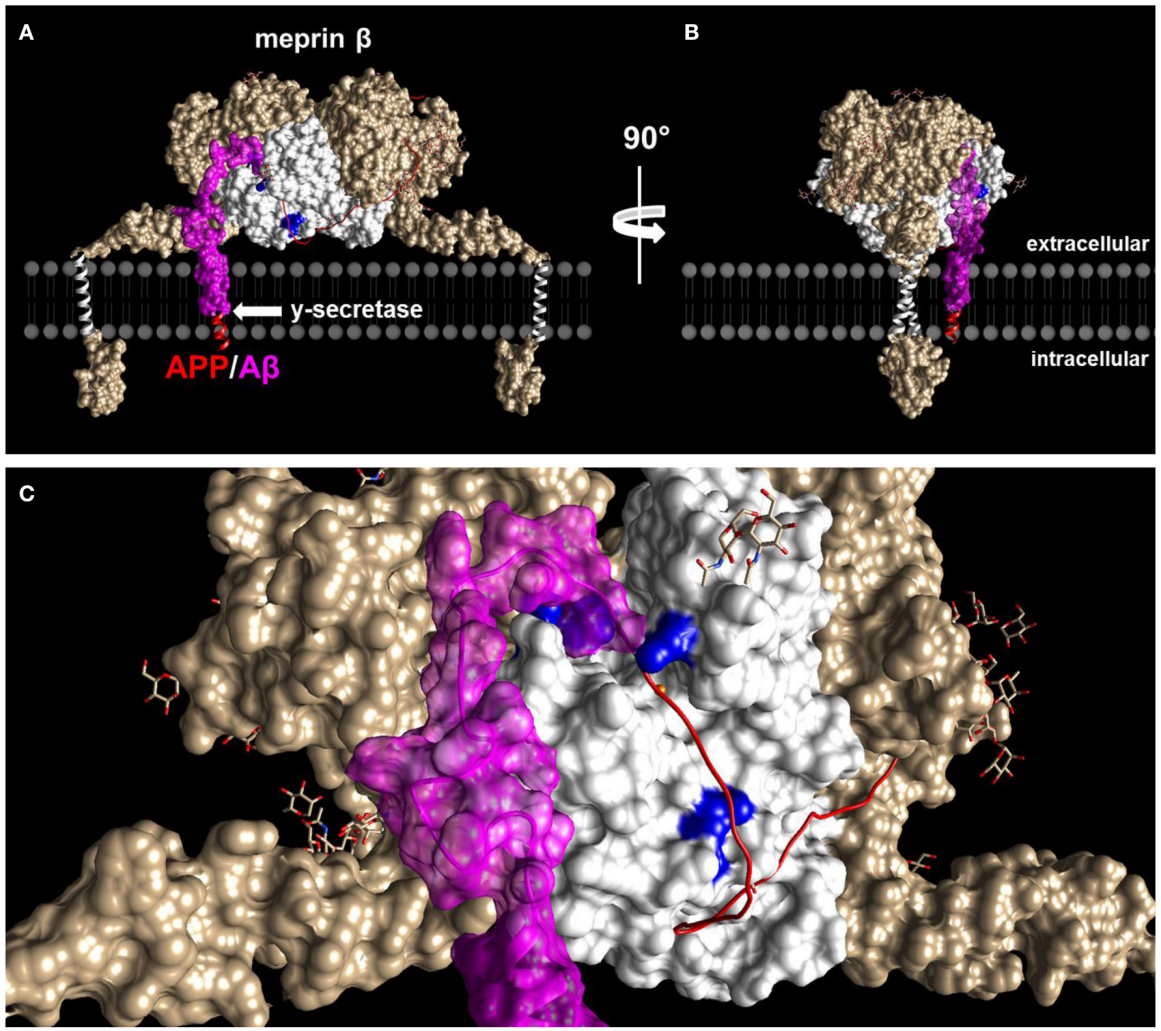
**Structural features of meprin β and APP interaction. (A)** Model of dimeric membrane bound meprin β (white and brownish) based on the crystal structure of the ectodomain (PDB 4GWN) in complex with part of the APP (red/magenta). **(B)** As in **(A)** but turned by 90° to the right. **(C)** Close up of the active site cleft of meprin β as shown in **(A)**. Positively charged amino acid residues important for the cleavage specificity are highlighted in blue. Part of the APP that builds the Aβ peptide is displayed as surface model. Glycans in meprin β are depicted as stick models.

In the last years, more and more focus has been put on modified N-terminally truncated Aβ variants. Increased levels of Aβ2-42 were detected in AD brains (Wiltfang et al., 2001). This is in line with results showing decreased levels of Aβ2-42 in CSF of AD patients (Bibl et al., 2012). Since BACE-1 is not capable in directly generating this peptide, a suggested model for the emergence of N-terminal truncation is the subsequent cleavage of the N-terminus of BACE generated Aβ1-x by either Aβ degrading enzymes like insulin-degrading enzymes (IDE) or neprilysin or the aminopeptidase A (APA) (Arai et al., 1999; Wiltfang et al., 2001; Wang et al., 2006). A candidate directly generating N-terminally truncated Aβ independent of BACE-1 is the metalloprotease meprin β. Meprin β is a multi-domain type I transmembrane protein, member of the astacin family of zinc-endopeptidases that is predominantly present as a dimer at the cell surface (Arolas et al., 2012; Figure 2). In 2011 meprin β was introduced as an alternative enzyme involved in APP processing (Jefferson et al., 2011). In 2012, N-terminally truncated Aβ2-40 peptides generated by meprin β (Figure 1), dependent on subsequent cleavage of the γ-secretase, but independent of BACE-1, were detected in supernatants of overexpressing cells (Bien et al., 2012). Interestingly, increased mRNA levels of meprin β were measured in AD brain homogenates supporting a potential role for this enzyme in neurodegeneration. Various posttranslational modifications of Aβ peptides have been described ranging from oxidation (Hou et al., 2002; Palmblad et al., 2002) to phosphorylation (Kumar et al., 2011, 2012), nitration (Kummer et al., 2011), glycosylation (Halim et al., 2011) or pyroglutamation of Glu3 of Aβ3-40 (Russo et al., 2002; Wittnam et al., 2012). These modifications have been shown to have an effect on the properties of the peptide. The oxidation at Met35 for example impedes the formation of protofibrils and fibrils from monomers (Hou et al., 2002). Nitration and pyroglutamation both increase the aggregation of Aβ (Schilling et al., 2004; Kummer et al., 2011). Meprin β was demonstrated to cleave APP at p3 position in a peptide derived *in vitro* assay (Bien et al., 2012), which would eventually lead to the release of Aβ3-40 peptides, containing an N-terminal pyroglumate modification. This cleavage site for meprin β, however, was so far only found in peptide cleavage assays and not in coexpression experiments with full length APP in cellular systems (Bien et al., 2012).

Several mutations within the APP sequence have been shown to have an impact on β-secretase cleavage by BACE-1. The recently described APP mutation A673T that has been shown to protect against AD as well as against cognitive decline in the elderly independent of AD was analyzed (Jonsson et al., 2012). The mutation is located at p2 of Aβ (Aβ-A/T) and has been shown to reduce BACE-1 mediated Aβ generation by 40% using synthetic peptides as substrates. Moreover, a significantly decreased Aβ production in human APP A673T-overexpressing primary neurons has been observed (Benilova et al., 2014; Maloney et al., 2014). Additionally, a decreased aggregation propensity of Aβ-A/T could be measured, which is showing the complexity of the protective effects of the substitution. As meprin β was shown to be involved in APP processing close to the BACE-1 cleavage site Schoenherr and colleagues investigated the effect of the APP A673T mutation on meprin β activity (Schönherr et al., 2016). The authors revealed a significant decrease of ~70% in the Aβ2-40/1-40 ratio compared to wildtype APP sequence in meprin β transfected cells and in a peptide cleavage assay using the APP A673T constructs. The decreased cleavage of APP by meprin β in the presence of the A673T substitution can nicely be explained by the cleavage preference of meprin β revealed by proteomics (Becker-Pauly et al., 2011). Here, a preference of alanine over threonine in P1' position was observed. As the activity of meprin β on APP processing varies with mutations around the original BACE-1 cleavage site Schoenherr and colleagues investigated whether the Swedish mutation of APP (K670N/M671L; APPswe) may affect meprin β cleavage activity. Surprisingly, Aβ2-x variants were completely missing in cells overexpressing meprin β and APP bearing the Swedish double mutation K670N/M671L (APPswe) which is located in close vicinity of the β-secretase cleavage site (Figure 1). This clearly shows a significant influence of amino acid substitutions around the β-secretase cleavage site for meprin β mediated Aβ generation.

Although BACE-1 is clearly the most prominent enzyme responsible for the generation of Aβ1-40 and Aβ1-42 peptides from the APP wildtype or APPswe sequences, meprin β may be responsible for generating small amounts of N-terminal truncated Aβ2-40 and Aβ2-42 peptides. N-terminal truncated Aβ peptides are almost exclusively generated by meprin β from the complete APP wildtype sequences or from APP carrying familiar Alzheimer disease mutations at the γ–secretase cleavage site but bearing the wildtype sequence around the β-cleavage site.

## AD mouse models

To analyze AD in an *in vivo* situation, different mouse models were already generated in the 1990's. However, these mouse models always show potential weaknesses which have to be considered before translating the results obtained from the mouse studies into the human situation. The major drawback is that cleavage of endogenous murine APP via the amyloidogenic pathway was never observed to lead to an AD-like phenotype. Hence, overexpression of different human APP forms in mice was and still is the most promising way to establish appropriate animal models. There are common models to study Aβ plaque pathology that all bear the APP Swedish mutation, such as 5xFAD mice, carrying mutations in the APP and PSEN1 genes [APP K670N/M671L (Swedish), APP I716V (Florida), APP V717I (London), PSEN1 M146L, and PSEN1 L286V; (Oakley et al., 2006)], J20 mice, carrying mutations only in the APP gene [K670N/M671L (Swedish) and the APP V717F (Indiana; Mucke et al., 2000)], or the 3xTg mice, carrying mutations in the APP, PSEN1, and the MAPT genes [K670N/M671L (Swedish), MAPT P301L, and PSEN1 M146V; (Oddo et al., 2003)]. These models all manifest an amyloid pathology although varying between animal models as well as differential learning and memory deficits. Thus, they appear to be appropriate models to mimic AD phenotypes at first sight. Notably, the human sequence of the Swedish familiar Alzheimer disease mutation (APPswe) is used in almost all AD animal models as it serves as a better substrate for BACE-1, thereby increasing production of total Aβ and specifically 1-X Aβ peptides (Citron et al., 1992; Cai et al., 1993). However, in light of the result put forward by Schoenherr and colleagues Aβ2-42 peptides which have been detected in brains of AD patients will not be generated in these mouse models. Therefore, it is likely that the actual effect of meprin β has been overlocked in many studies focusing on APP processing. This issue must be considered when analyzing the results from the ongoing clinical trials, using BACE-1 inhibitors for the treatment of AD patients.

## Meprin β and APP beyond AD

As mentioned above in it has been shown that meprin β additionally cleaves APP apart from the Aβ sequence resulting in N-terminal APP fragments (NTF) (Jefferson et al., 2011). These fragments were also detected in human brain homogenates suggesting that this interaction not only occurs in overexpressing cell systems, but probably also under endogenous levels in the human brain. The *in vivo* relevance for this proteolytic event was further supported by analyzing brain lysates from meprin β deficient mice where this particular N-APP cleavage was abolished (Jefferson et al., 2011). Interestingly, Tessier-Lavigne and colleagues showed that an N-terminal APP fragment found in AD patients binds the *death receptor 6* (DR6) thereby inducing neurodegeneration (Nikolaev et al., 2009). Thus, it was speculated whether meprin β might be the responsible protease in this regard. However, purification and characterization of the meprin β generated N-APP fragments showed neither negative nor positive influence on neuronal cell viability (Jefferson et al., 2011). Therefore, it is likely that APP cleavage by meprin β in the N-terminal region has rather protective function.

## Physiological functions of meprin β

Meprin β is strongly expressed in the intestinal epithelium and in kidney proximal tubular cells, and to minor levels in several other tissues, e.g., in skin, certain immune cells, and the brain (Broder et al., 2013). Besides many potential substrates analyzed *in vitro* only few *in vivo* functions of meprin β have been reported so far. In the intestine, where meprin β is found at the apical site of epithelial cells, the protease is responsible for the detachment of the mucus by cleaving mucin 2, an important step for proper barrier function (Schütte et al., 2014). Along the same line, meprin β cleaves type 1 pili of adherent-invasive *E. coli*, thereby preventing colonization of these bacteria in the ileal mucosa of Crohn's disease patients (Vazeille et al., 2011). Several other studies provide evidence for an important immunological function of meprin β (Banerjee and Bond, 2008; Bylander et al., 2008; Banerjee et al., 2009, 2011; Yura et al., 2009; Broder and Becker-Pauly, 2013; Zhang et al., 2015). As known for other members of the astacin family, namely BMP-1 (bone morphogenetic protein 1) and tolloids, meprin β is involved in the maturation of procollagens I and III (Kronenberg et al., 2010; Broder et al., 2013; Prox et al., 2015). Collagen, the most abundant protein in human body, is a crucial factor for the integrity of connective tissue, tendon, and bone. To prevent fibril assembly already in intracellular compartments, collagens contain C- and N-terminal prodomains that need to be removed proteolytically by extracellular proteases. Meprin β is such an enzyme, and *Mep1b*^−/−^ mice show severe impairments of the connective tissue in skin characterized by reduced tensile strength and decreased collagen deposition (Broder et al., 2013). On the other hand, under pathological conditions, overexpression of meprin β is associated with fibrotic diseases, such as keloids of the skin (Kronenberg et al., 2010) and pulmonary hypertension (PH) (Biasin et al., 2014). PH is a severe fibrotic condition of the lung with very bad prognosis for the patients that die 2–3 years after diagnosis. In genetic screens of lung tissues from patients and a mouse model of PH meprin β was found amongst the most up-regulated genes (Biasin et al., 2014). Here, AP-1 transcription factor complex was identified as an inducer of *Mep1b* mRNA expression. Whether meprin β is only involved in the progression of fibrosis by collagen maturation and deposition, or if the protease also contributes to the onset of the disease as a pro-inflammatory enzyme has to be further investigated.

## Regulation of meprin β

As meprin β associated pathologies, such as fibrosis, cancer, and AD, are mostly based on increased expression and activity of the protease, information about the regulation of the enzyme is important.

## Activation

Meprin β is expressed as an inactive zymogen and requires proteolytic removal of its propeptide to gain full enzymatic activity. Several tryptic serine proteases have been identified as activators of latent meprin β, amongst them kallikreins (KLKs) 4, 5, and 8, as well as pancreatic trypsin (Ohler et al., 2010). The latter is supposed to be the physiological activator in the intestine, thereby contributing to the mucus-cleaving activity of meprin β (Schütte et al., 2014), whereas KLKs may rather be important in skin and mesenchymal tissues (Ohler et al., 2010). Based on the crystal structure of the ectodomain of human meprin β it became evident that the activation site at amino acid position Arg61 is in very close proximity to the cell surface (Arolas et al., 2012). Therefore, it was doubtful whether the previously described soluble tryptic activators, which were identified in *in vitro* assays using recombinant soluble promeprin β, are capable of activating the membrane bound meprin β. Indeed, not even trypsin was able to cleave off the propeptide of full length meprin β, which led to the assumption that possible candidates are most likely membrane bound serine proteases. In this regard, matriptase-2 (MT-2), a type 2 transmembrane protein, was found to fully activate meprin β at the cell surface (Jäckle et al., 2015). Consequently, MT-2 mediated activation of meprin β resulted in increased APP shedding and subsequently decreased sAPPα levels. If this proteolytic interaction may have impact on neurodegenerative disorders has to be shown. Surprisingly, however, in a different study MT-2 was found to directly cleave neuronal APP695, but was effectively inhibited by the Kunitz protease inhibitor (KPI) domain present in other APP isoforms (APP751 and APP770) from the periphery (Beckmann et al., 2016). Of note, the additional domains in APP751 (KPI) and APP770 (KPI/OX2) do not lead to altered proteolytic processing by meprin β (Jefferson et al., 2011). This demonstrates how complex the proteolytic processing of APP can be and how important it is to elucidate the time-dependent and site-specific cleavage events with regard to the different proteases, such as ADAM10, BACE-1, meprin β, or MT-2.

## Inhibition

The tissue inhibitors of metalloproteinases (TIMPs) are effective regulators of the catalytic activity of matrix metalloproteases (MMPs) and ADAMs (Yamamoto et al., 2015). TIMPs, however, do not inhibit meprin β, and so far only one rather unspecific endogenous inhibitor was identified, namely fetuin-A (Kruse et al., 2004; Hedrich et al., 2010). Interestingly, calcium was found to inhibit the proteolytic activity of meprin β by binding to a cluster of negatively charged amino acids in close proximity to the active site, thereby inducing conformational changes (Arnold et al., 2015). However, the inhibition constant of calcium for meprin β is about 11 mM, which resembles the concentration in the endoplasmic reticulum and not at the cell surface. The amino acid residues forming the calcium binding site in meprin β contribute to correct folding of the protease. Mutations within the calcium binding site resulted in protein that stacks to the ER and is not properly secreted (Arnold et al., 2015). The calcium concentration needed for the inhibition of meprin β is rather not relevant for extracellular inhibition, at least under physiological conditions. Thus, regulation of meprin β's activity must occur on a different level. One possibility is the ectodomain shedding of meprin β by ADAM10 or ADAM17 (Jefferson et al., 2013). Very importantly, only membrane bound meprin β is capable of generating aggregation prone Aβ2-x peptides and not the shed solubilized protease (Bien et al., 2012). Therefore, ADAM10 does not only prevent Aβ release by cleaving APP at the α-secretase site, but additionally by the shedding of meprin β and thereby preventing its activity toward the β-secretase site.

## Localization

As mentioned above, shedding of APP by meprin β occurs predominantly at the cell surface and thus competes with ADAM10 for the substrate (Schönherr et al., 2016). Recent studies demonstrated that ADAM10 localization and maturation is influenced by tetraspanins (TSPANs), building microdomains of protein clusters at the cell surface (Prox et al., 2012). In a yeast-two-hybrid approach TSPAN8 was identified as an interaction partner of meprin β, which was further proven by split-RFP and luciferase complementation assays (Schmidt et al., 2016). It was further demonstrated that APP together with meprin β is located in TSPAN8 enriched microdomains. However, overexpression of TSPAN8 had no obvious influence on meprin β activity and APP cleavage. Nevertheless, orchestration of proteases and substrates at the cell surface by regulatory factors has to be further studied to fully understand the complex proteolytic processing of APP by different enzymes.

Concluding, the protease meprin β appears as an important candidate for further studies on APP processing and Aβ generation and may have a contributing role to the physiological and pathophysiological function of APP itself.

## Author contributions

All authors (CP, CB) contributed to the concept and drafting the work. The authors ensure that questions related to the accuracy or integrity of any part of the work are appropriately investigated and resolved.

## Funding

This work was supported by the Deutsche Forschungsgemeinschaft, grant BE 4086/2-2 (to CB), and grant PI 379 6-2 (to CP).

### Conflict of interest statement

The authors declare that the research was conducted in the absence of any commercial or financial relationships that could be construed as a potential conflict of interest.
